# Optimal locomotor strategy for predator avoidance in fish prey

**DOI:** 10.1242/jeb.249595

**Published:** 2026-06-12

**Authors:** Ashley N. Peterson, Matthew J. McHenry

**Affiliations:** Department of Ecology and Evolutionary Biology, University of California, Irvine, 321 Steinhaus Hall, Irvine, CA 92697, USA

**Keywords:** Predator–prey interactions, Avoidance strategy, Agent-based modeling

## Abstract

The control and mechanics of locomotion determine the outcome of a variety of predator–prey interactions. The strategies that optimize prey survival have primarily been considered for cases where the prey initiates an escape in response to a predator's approach. However, a diversity of prey survive by avoiding encounters with predators without the aid of evasive maneuvers. To understand locomotor strategies for these conditions, we developed an agent-based numerical model for individual prey damselfish (*Chromis viridis*) when targeted by a lionfish predator (*Pterois volitans*). Based on previous experiments, we modeled the predator to track prey with a pure-pursuit strategy and the prey avoided the predator with intermittent bouts of swimming that were biased away from the threat. We found that simulations were statistically similar to experimental measurements in the duration of the interactions. In contrast to distance-optimal evasion models, a sensitivity analysis revealed that prey survived encounters with faster predators most successfully by swimming towards the predator and capitalizing on the relatively slow capacity of the predator to change direction. These findings demonstrate the power of agent-based mathematical modeling to determine the salient features that determine the outcome of coupled predator–prey interactions.

## Teaser

**Summary:** Agent-based modeling suggests that prey can enhance their survival by employing a range of avoidance strategies, particularly by biasing locomotion toward fast predators.

## INTRODUCTION

Locomotion plays a critical role in a diverse array of predator–prey interactions. Although the coupled nature of these interactions is broadly appreciated (e.g. Jackson, 1990; Shine and Sun, 2003; Alfaro, 2002; Slip and Shine, 1988; deVries et al., 2012; Pavlov and Kasumyan, 2002; Price and Mensinger, 1999; Cooper et al., 1985), their complexity often challenges the ability of experimentalists to identify the salient sensorimotor traits that determine prey escape or capture (Milton and Bergmann, 2023). Theoretical models offer a framework for resolving the strategic aspects of predator–prey interactions and a significant contingent of these models have focused on strategy in fish. Fish–fish interactions can be constrained to two-dimensional motion and may be studied in a laboratory, where the trajectories of predator and prey can be measured with high precision and compared against the predictions of mathematical models (e.g. Domenici and Blake, 1993; Peterson and McHenry, 2022; McKee and McHenry, 2020; Nair et al., 2017; Soto and McHenry, 2020; Stewart et al., 2013; Kane and Higham, 2014; Domenici et al., 2011; Weihs and Webb, 1984; Soto et al., 2015; McHenry et al., 2019; Free et al., 2019; Thompson et al., 2023; Domenici, 2002).

The pursuit strategy of predators has been evaluated by comparing kinematic measurements against the predictions of agent-based models. This approach was developed to understand targeting and pursuit behaviors in hawks, bats, robber flies and dragonflies (Fabian et al., 2018; Brighton et al., 2017; Kane et al., 2015; Kane and Zamani, 2014; Olberg, 2012; Ghose et al., 2006; Howland, 1974) and has since been adapted to fish predators. Research on bluefish (*Pomatomus saltatrix*: McHenry et al., 2019; Free et al., 2019) and blue acaras (*Andinoacara pulcher*: Szopa-Comley and Ioannou, 2022) suggests that fish pursue the instantaneous position of their prey with varying forms of a strategy known as ‘pure pursuit’ (Isaacs, 1965; Weihs and Webb, 1984). Blue acaras maintain a bearing near zero, while bluefish employ deviated pursuit, where the predator directs its heading toward the prey with a small angular offset. Therefore, fish predators appear to operate under a relatively simple behavioral algorithm to close the distance to their target.

Considerations of prey strategy have largely focused on the direction of an escape response when initiated by a predator's approach. The optimal direction to maximize the distance from the predator may be formulated from classic game-theoretical models, under the assumption of a fixed heading by both animals (Isaacs, 1965; Weihs and Webb, 1984; Domenici, 2002; Soto et al., 2015; Kawabata et al., 2023; Martin et al., 2022). This strategy depends on the speed of both animals, with the direction mattering most critically when the prey is only slightly slower than the predator. Experimental tests have shown that the distance-optimal strategy is predictive of the direction of escape responses by fish larvae (Jiao et al., 2023) and is consistent with the behavior of many adult fish prey (Domenici et al., 2011).

While escape responses can play an important role in survival, fish may alternatively survive merely by avoiding encounters with predators. Avoidance may be particularly effective against predators such as lionfish, which are slower than their prey (Peterson and McHenry, 2022). Reef fishes, such as damselfish, forage by intermittent bouts of swimming and may cohabitate with lionfish by maintaining a safe distance. Despite the prey possessing a speed advantage during routine locomotion, lionfish have proven to be voracious and invasive predators (Albins, 2015; Côté and Smith, 2018). They succeed not with speed but with a ‘persistent pure-pursuit’ strategy where they doggedly pursue prey until executing a highly effective suction-feeding strike on the occasions where the prey fails to maintain vigilance (Peterson and McHenry, 2022). Under these conditions, the lionfish's strike effectively prevents the opportunity for an escape response by the prey. By moving slower than their prey, lionfish violate a key assumption of the classic evasion models. That feature, combined with the stochastic nature of prey foraging behavior, requires a novel analytical framework to understand the role of avoidance strategy in predator–prey interactions.

In the present study, we developed a mathematical model of locomotion to evaluate the aspects of avoidance behavior that matter for survival and to identify optimal strategies. Specifically, we used an agent-based model to simulate the movement of predator and prey using behavioral rules that were parameterized with experimental measurements of their kinematics. We parameterized and validated the model by comparison with kinematic measurements from previous experiments of lionfish (*Pterois volitans*) as they preyed upon damselfish (*Chromis viridis*) (Peterson and McHenry, 2022). The model formulated predictions of locomotion that were based on the assumption that each fish was capable of sensing the position of the other. The predator moved at a fixed speed, with a heading determined by pure pursuit. The prey avoided the predator by swimming in a direction that was determined by its particular avoidance strategy, while also exhibiting intermittent bouts of routine swimming. We modeled the stochastic nature of this routine swimming by incorporating a Monte-Carlo approach by use of random-number generation for some kinematic parameters. We thereby performed a series of simulations to generate populations of results that we tested against the previous experiments (Peterson and McHenry, 2022). We then leveraged our validated model to perform sensitivity analyses that evaluated the effects of behavioral parameters across a range of predator speeds. These results for avoidance locomotion were compared with the predictions of classic evasion models.

**Table:** 

**List of symbols and abbreviations**	
*a*	prey acceleration
*d*_wall_	distance to wall
*K*_avoid_	avoidance constant
*K*_track_	tracking constant
*K*_turn_	wall turning constant
*K*_wall_	wall avoidance constant
*P*_strike_	strike probability
*S*_behav_	prey speed
*S*_behav,0_	initial prey speed
*S*_pred_	predator speed
*t*	time
*t*_behav,0_	behavior start time
*T*_accel_	acceleration duration
*T*_decel_	deceleration duration
*T*_interact_	interaction duration
α	angle of the line of sight
β	relative heading
β_0_	avoidance heading angle
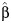	routine heading constant
γ	angular position
θ_pred_	heading angle of predator
θ_prey_	heading angle of prey
θ_tan_	wall tangent angle
θ_tan,0_	wall bias angle
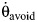	rate of turning for predator avoidance
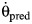	rate of turning in predator
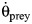	rate of turning in prey
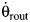	rate of routine turning in prey
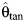	wall error offset angle
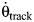	rate of turning in tracking
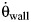	rate of routine turning for wall avoidance
τ	decay constant
φ	bearing
φ_0_	bearing set-point

## MATHEMATICAL MODEL

Both predator and prey were modeled as particles with 2D motion governed by first-order ordinary differential equations and bounded by a circular arena. Numerical solutions to these equations predicted the trajectories of the two animals, given their initial positions and behavioral parameter values. In this section, we detail the relevant equations for the predator and prey, the method for parameterizing the model with experimental measurements, and the procedure for obtaining the numerical solutions.

### Modeling the predator

Lionfish move at a consistent speed, but adjust their heading continuously towards their prey (Peterson and McHenry, 2022). We therefore modeled the predator speed (*S*_pred_) as a fixed parameter with respect to time. In order to replicate observed behavior, we devised a controller that avoids traversing the walls of the circular area while lionfish attempt to target the prey with pure pursuit. This was achieved by calculating the rate of heading change for the predator (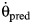) as the sum of terms that serve to avoid the walls (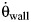) and track the prey (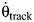):
(1)

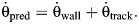
We modeled wall avoidance by increasing their turn rate exponentially as the distance to the wall (*d*_wall_) approached zero, as given by the following relationship:
(2)

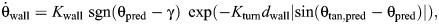
where θ_pred_ is the instantaneous heading of the predator, *K*_wall_ and *K*_turn_ are constants that determine the intensity of wall avoidance, γ is the angular position of the fish with respect to the arena's center, and θ_tan_ is the angle tangent to the arena's boundary. The tangent angle for the predator was calculated as follows:
(3)

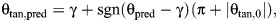
where θ_tan,0_ is the offset angle from which to turn away from the wall.

**Fig. 1.**
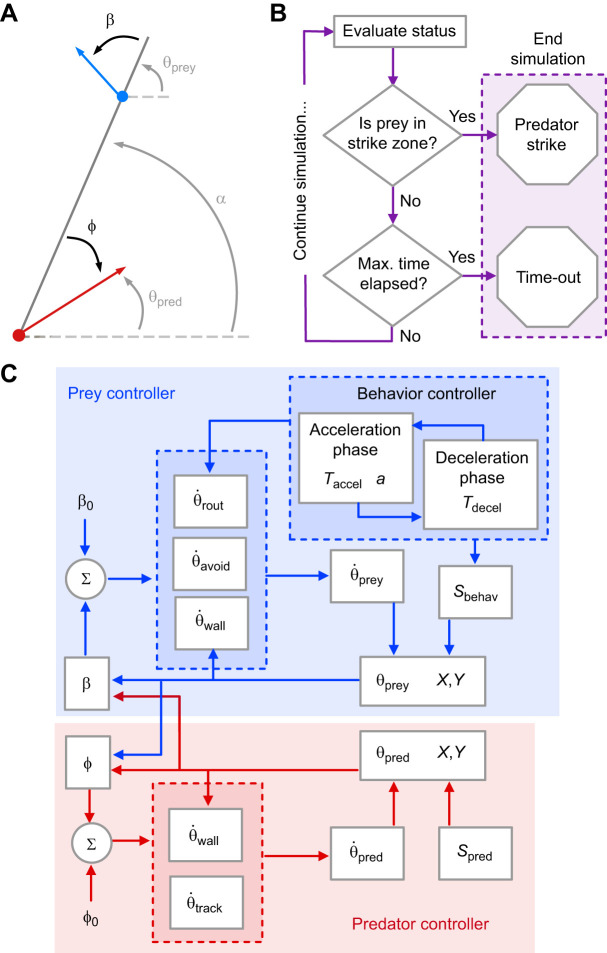
**Schematic illustrations of the agent-based predator–prey model.** (A) Depiction of angles used for the control of simulated predator and prey, with the position and direction of the predator (in red) and prey (in blue) and the heading (θ, arrows), defined relative to the global frame of reference (dashed lines). The line of sight (gray line, at angle α) represents the prey's angular position relative to the predator. Angles related to pursuit (the bearing angle, φ) and avoidance (the relative heading angle, β) are defined with respect to the line of sight. (B) Control of simulation termination (filled purple rectangle) is depicted by a decision tree. At the end of each time step, the simulation clock and position of the predator and prey were evaluated. Strike-terminated simulations occurred when prey were within the strike zone of a predator. Simulations that exceeded the maximum set duration (1200 s) were time terminated. (C) A diagram of control for simulated predator–prey behaviors, with the control of the simulated predator (red arrows, Eqns 1–5) and the prey (blue arrows, Eqns 6–9). Prey locomotor behavior (behavior controller) included randomly generated parameter values determined at the beginning of a behavioral phase (Eqns 10–12, see List of symbols and abbreviations for definitions).

To model pure pursuit, the simulated predator attempted to maintain a zero bearing (φ_0_) by modulating the rate of change in heading due to tracking, as determined by the following relationship (Shneydor, 1998):
(4)

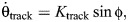
where *K*_track_ is the tracking constant. The magnitude of *K*_track_ represents a measure of sensorimotor delay that determines the responsiveness of the predator to movement by the prey. The instantaneous bearing angle was calculated as follows (Shneydor, 1998):
(5)

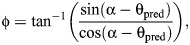
where α is the prey's angular position relative to the predator and θ_pred_ is the predator's heading angle (Fig. 1A). The occurrence of a strike was dependent upon both the distance between the predator and prey and the angular position of prey with respect to the line of sight, a combination which we term the ‘strike zone’. Based on previous observations of lionfish striking behavior (Peterson and McHenry, 2022), we determined the threshold range of the bearing angle for a strike to occur within −15 deg<φ<15 deg. The strike distance varied between 2 and 20 cm, depending on the type of simulation we were running (detailed below).

### Modeling the prey

The prey's behavior was modeled through factors affecting changes in speed and heading that occur during intermittent bouts of swimming. This was achieved with a controller that included a combination of fixed parameters and time-variable parameters that changed within a simulation. Values for both types of parameter (Tables S1 and S2) were based on previous measurements (Peterson and McHenry, 2022). While fixed parameter values remained constant throughout a simulation, time-variable parameters were determined at the beginning of a behavioral phase (an acceleration or deceleration) and remained constant until the end of that phase.

Speed was controlled by modeling phases of acceleration and deceleration. Parameter values for the magnitude of acceleration (*a*), the duration of acceleration (*T*_accel_) and the duration of deceleration (*T*_decel_) were randomly generated before each phase, based on probability density functions (PDFs) fitted to previous measurements (Peterson and McHenry, 2022). For acceleration phases, we calculated the speed at time *t* (*S*_accel_) by assuming a constant acceleration:
(6)

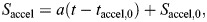
where *a* is acceleration magnitude, *t*_accel,0_ and *S*_accel,0_ are respectively the time and speed at the initiation of a bout of acceleration. The speed values during simulations were not permitted to exceed the maximum of measured values (40 cm s^−1^). Over the periods of deceleration (for a duration of *T*_decel_), the speed (*S*_decel_) was modeled as a decay function, as follows:
(7)

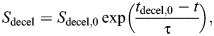
where *t*_decel,0_ and *S*_decel,0_ are respectively the time and speed at the start of the deceleration period and τ is the decay constant.

Heading changes were governed by the tank walls (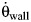), avoidance of the predator (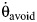) and routine changes in direction (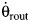). Hence, we modeled the rate of change in a prey's heading (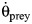) as the sum of these factors:
(8)

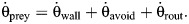
The turning rate from the walls was calculated in the same manner as for the predator (Eqn 2), but the tangent angle was determined differently to account for spontaneous change in direction near the walls. This tangent angle was consequently found by the following equation:
(9)

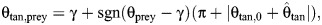
where 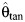 is a randomly generated bias angle for the prey. Predator avoidance was calculated as follows:
(10)

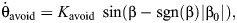
where β is the prey's relative heading (Fig. 1A), and *K*_avoid_ is the avoidance constant. This metric dictates the level of responsiveness of the controller and much like the tracking constant of the predator, *K*_avoid_ introduces a sensorimotor delay that determines the magnitude of predator avoidance. The avoidance heading angle, β_0_, is equivalent to the set-point for the control of predator avoidance, which dictates the direction in which the prey aims to avoid the predator. A zero value is directed away from a predator and 180 deg is toward the predator. The relative heading (β) is the prey's orientation relative to the line of sight (Fig. 1A), given by the following relationship:
(11)

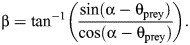
Routine turns were initiated at the start of each new period of acceleration or deceleration and were determined by the following equation:
(12)

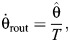
where *T* is the duration of the period of acceleration (*T*_accel_) or deceleration (*T*_decel_), and 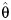 is a randomly generated angle, from which the rate of routine turning (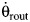) was found. The angle 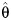 was chosen at the beginning of each acceleration and deceleration phase (‘circ_vmrnd’ function in MATLAB; Berens, 2009) from a von Mises distribution with an average direction of 0 deg. The degree to which a value of 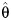 was concentrated around zero was determined by the routine heading constant (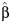) (Fig. S1A), which was fixed at the onset of a batch of simulations. For example, there was an equal probability that the value of a randomly generated angle fell between 0 and 360 deg when the routine heading constant was zero (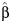
=0). Angular values of 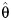 and the duration of *T* served to modulate the magnitude of changes in heading. A 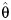 value of zero would result in little influence in the rate of turning from routine swimming (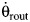), while values of 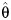 close to 180 deg would heavily influence the overall rate of turning.

### Numerical simulations

Our agent-based model necessitated a numerical solver to simulate the interactions between predator and prey (Fig. 1C). This was achieved by integrating the first-order differential equation for the predator's heading (Eqn 1), and the prey's heading (Eqn 8) and speed (Eqns 6 and 7). All simulated predator–prey interactions assumed a fixed diameter (1.2 m) for the arena, equal to the size of previous experiments (Peterson and McHenry, 2022). The initial conditions and parameter values used in a particular simulation depended on whether we were attempting to validate the model or were performing a sensitivity analysis, as described below. Integration was performed with a variable time-step solver, based on an explicit fourth-order Runge–Kutta formulation (the ‘ode45’ function in MATLAB, v.2021a, Mathworks, Natick, MA, USA). A simulation ended when one of the two termination conditions occurred: either the prey entered within the strike zone of the predator or the duration of the simulation exceeded the predetermined maximum duration (Fig. 1B). Each simulation that ended after exceeding the maximum duration was deemed ‘time terminated’, while those that ended with the prey reaching the strike zone were designated as ‘strike terminated’. A number of kinematic parameters were used for all simulations that were determined by trial-and-error inspection of results to qualitatively match kinematic measurements (itemized in Table S2). Each simulation was evaluated in terms of the interaction duration (*T*_interact_) between predator and prey and the strike probability (*P*_strike_).

## MODEL VALIDATION

We validated our model with two types of tests. Our ‘experiment-specific validation’ tested whether the model could replicate the interactions measured in individual experiments (Peterson and McHenry, 2022), using particular measured kinematic parameters. The ‘population validation’ evaluated the model's ability to replicate average metrics of the behavior of prey at the population level. These validation procedures were performed by running batches of simulations. Each batch consisted of 300 simulations to generate populations of predicted outcomes with variation generated by randomly selected parameter values. The overall probability of a predator strike within a batch of simulations could be determined by finding the proportion of strike-terminated simulations. Finally, we compared the mean duration of batches of simulated interactions against the measured duration of the experiments.

### Experiment-specific validation

The simulations for the experiment-specific validation used fixed variable values that were drawn from measurements specific to each experiment. This included the initial position and heading of each fish and the lionfish's strike distance. The predator was modeled with a constant speed, equal to the mean value of the experimental measurements. The maximum duration (300 s) used for experiment-specific validation corresponds to the period of previous measurements (Peterson and McHenry, 2022). The prey's changes in speed were determined by random-number generation from PDFs fitted to the values from within each experiment for acceleration magnitude, acceleration duration and deceleration duration (Eqns 6 and 7). The only randomly selected variable in experiment-specific validations was 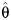, which contributed to the rate of turning in routine swimming (Eqn 12; Fig. S1A), which was generated at the start of each period of acceleration and deceleration.

We compared the population of predicted outcomes generated by our model against the results of 17 previous experiments (Peterson and McHenry, 2022). The percentage of strikes measured in experiments (82%) fell within one standard deviation of the mean proportion of strike-terminated simulations (72±36%). The mean duration of strike-terminated simulations was not significantly different from the duration of experiments that ended in a strike (*P*=0.67, Wilcoxon sign rank test, *N*=30; Fig. S2A). For the purposes of validation, we removed time-terminated simulations from this analysis because of the artificial limitations we placed on the maximum duration. Therefore, the model simulations largely predicted the duration of our experimental results.

### Population validation

**Fig. 2.**
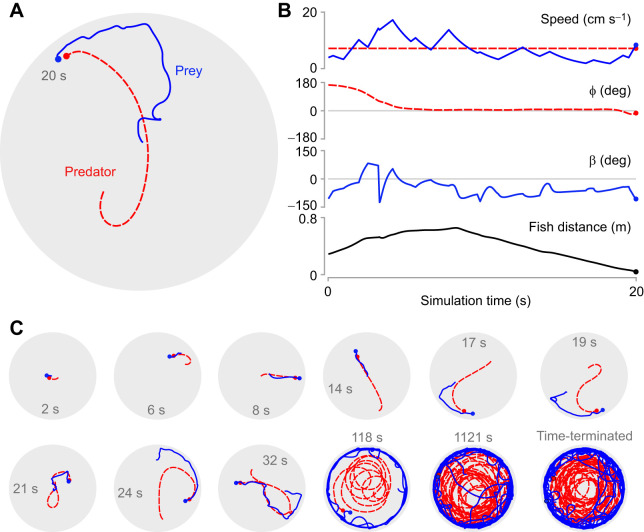
**Simulated trajectories of predator and prey fish.** (A) An example of the trajectories of predator (dashed red line) and prey (blue line) from a single simulation where ‘population validation’ values were used (see Tables S1 and S2). The gray circle depicts the arena boundaries. The final position of the predator and prey is depicted by the red and blue circles, respectively. (B) Time series data of predator (red dashed line) and prey speed (blue line), predator bearing angle (ϕ, red dashed line), prey avoidance heading (β, blue line) and the predator–prey distance (black line) are depicted for the example simulation in A. The model controller setpoint values for the prey avoidance heading and predator bearing angle are depicted by gray horizontal lines. (C) To illustrate the unique nature of individual simulations within a batch, further examples of predator–prey trajectories modelled using ‘population validation’ values are depicted, with the corresponding simulation duration. All example simulations, except for a single ‘time-terminated’ simulation, ended with the predator striking the prey after the specified duration.

Because the population validation was not focused on any particular experiment, these simulations employed a number of variable and parameter values that were selected randomly. The random variables included the initial conditions for the position and heading of predator and prey, each with an equal probability for each position in the arena and body orientation (see Fig. 2 for example model trajectories and kinematics). The strike distance of the predator was also chosen at the beginning of each simulation from a ‘log-normal’ PDF fit (using the ‘makedist’ function in MATLAB) to experimental measurements (Fig. S1B, Table S3). We additionally fitted PDFs to the experimental mean and standard deviation values of parameters that determined the speed of prey (α, *T*_accel_ and *T*_decel_; Eqns 6 and 7; Table S2), such that each parameter drew from distributions with the mean and standard deviation of experimental data. These PDFs provided the values for a simulation-specific distribution for each parameter, which were created using a randomly chosen mean and standard deviation at the onset of each simulation. We found that the mean strike probability of simulations (69% and 80%, when the maximum duration was 300 s and 1200 s, respectively) was slightly less than the percentage of strikes within experiments (82%). However, the mean duration of experiments where strikes occurred was not significantly different from strike-terminated simulations in batches, regardless of the maximum interaction duration (*P*=0.45 and *P*=0.09, when the maximum duration was respectively 300 s and 1200 s; Fig. S2B).

## RESULTS

We performed a series of sensitivity analyses that considered the effect of individual parameters on the outcome of predator–prey interactions. Specifically, we examined individual components that contribute to the anti-predator behavior of prey. The nature of each parameter dictated the method in which it was varied. For parameter values that did not depend on random number generation, values were varied across batches of simulations. In contrast, prey swimming behavior relied on random numbers within each simulation from PDFs set at the onset of each simulation. We therefore performed sensitivity analyses for these parameters by altering the distributions from which the random numbers were drawn. In particular, at the onset of batches of simulations, the population mean of a particular parameter was changed in increments of ±10% with a constant standard deviation. Simulation distributions were bounded by the maximum and minimum measured values to maintain biological relevance. Finally, we ran a series of simulations for each combination of predator speed and prey parameters.

### Predator behavior

**Fig. 3.**
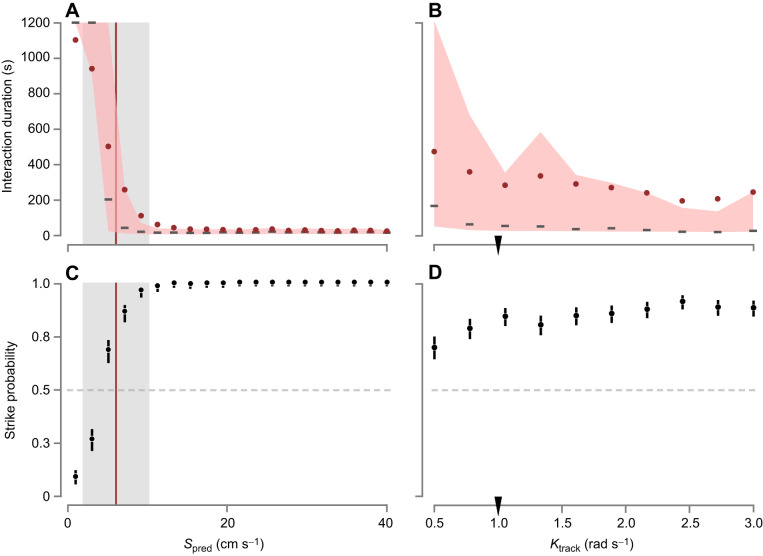
**Sensitivity analyses of predator parameters.** Predator speed (*S*_pred_; A,C) and the tracking constant (*K*_track_; B,D) were varied for batches of simulations. (A,B) The red shaded region depicts the bounds of the first and third quartiles of the interaction duration, with the median (horizontal gray bar) and mean (red circles) values shown for each parameter value along the *x*-axis. (A,C) The mean measured lionfish speed (vertical red line) ±1 s.d. (gray shaded region) (Peterson and McHenry, 2022). (C,D) The mean strike probability (gray circles) for each parameter value, with 95% confidence intervals (vertical black lines), relative to an even strike probability (dashed gray line). (B,D) The simulation validated value (black triangle) of the tracking constant is shown on the *x*-axis.

Our sensitivity analysis showed that changes in predator speed generated the most substantial effect on the outcome of predator–prey interactions. For example, a speed reduction of one standard deviation (1 s.d.) below the mean measured speed in experiments (6±4 cm s^−1^; Peterson and McHenry, 2022) represented nearly a 200% increase in the mean interaction duration. An equivalent increase in speed above the mean resulted in a 80% decline in the interaction duration (Fig. 3A). Increasing speed beyond the range of lionfish showed little effect, with the mean interaction duration declining asymptotically.

The strike probability exhibited a nearly inverse relationship to the interaction duration (Fig. 3C). The predator exhibited an almost ideal mean strike probability at the higher speeds. In contrast, less than 10% of simulations within a batch ended in a strike at the slowest predator speeds (<3 cm s^−1^). Therefore, a predator moving faster than lionfish would attain negligible benefits, but any slower speeds would yield drastically adverse consequences.

The tracking constant (*K*_track_) determined how quickly a predator adjusted its heading in response to a change in the prey's position (Eqn 4). We found only a modest decrease in the interaction duration across the range of tracking constant values considered, with the highest variation at lower values of the tracking constant (Fig. 3B). The mean value for the strike probability increased slightly (from 0.80 to 0.90) over this range (Fig. 3D). The greatest changes in interaction duration and strike probability occurred at tracking constant values below our estimate for lionfish (*K*_track_<1.0).

These results indicate that the lionfish operate at a critical strategic regime when interacting with prey. Slower, or less responsive, lionfish would be severely disadvantaged in their ability to capture prey. The converse does not hold true: increased speed or responsiveness many times above the estimated lionfish values would be required to realize only modest benefits for lionfish.

### Prey speed control

**Fig. 4.**
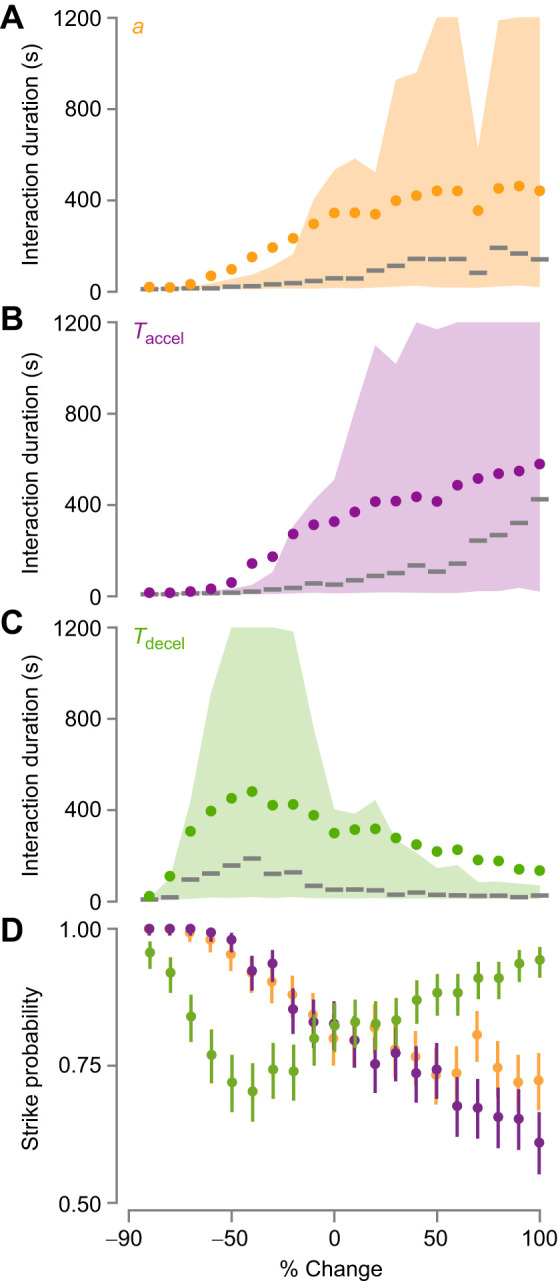
**Sensitivity analyses for the effects of prey swimming behavior.** The acceleration magnitude (*a*; A, orange), acceleration duration (*T*_accel_; B, purple) and deceleration duration (*T*_decel_; C, green) were varied as a function of the percentage change in the mean parameter values (*x*-axis). The mean parameter value was used to create probability density functions (PDFs), from which randomly generated values for each simulation were determined. (A–C) The shaded regions depict the bounds of the first and third quartiles of the interaction duration, with the median (horizontal gray bar) and mean (colored circles) values. (D) The variation in strike probability across simulations that varied the percentage change of acceleration magnitude (orange), acceleration duration (purple) and deceleration duration (green), with 95% confidence intervals (vertical lines).

We found that changes in the magnitude (*a*) or duration (*T*_accel_) of acceleration by prey yielded similar effects on the interaction duration and strike probability. Decreasing the magnitude of either to a low level (<−50% of the mean value) generated simulations of negligible duration (Fig. 4A,B), which almost always terminated with a predator strike (Fig. 4D). This is not surprising, given the slow swimming speed of the prey when acceleration is reduced to such low levels. Increasing the magnitude and duration of acceleration increased the speed of the prey, which were found to have more pronounced effects at low parameter values. For example, a 50% increase in acceleration magnitude caused only a small (100 s) increase in mean interaction duration while a 50% reduction caused the interaction duration to decline rapidly from ∼350 to ∼100 s (Fig. 4A). Nonetheless, monotonic increases in interaction duration and reductions in strike probability (Fig. 4D) were found across increases in acceleration magnitude and duration.

We considered the effects of the deceleration duration (*T*_decel_) on the outcome of interactions. Longer decelerations generated a monotonic decrease in the mean interaction duration (Fig. 4C) and increases in strike probability (Fig. 4D) over a broad range of values (from −50% to +100% change from the observed mean). However, shorter decelerations (from −90% to −50%) exhibited an opposite trend, where increases in duration generated an increase in interaction duration and a decrease in strike probability. Shortening the deceleration duration resulted in proportionately more frequent periods of acceleration, which created an increased interaction duration and a decreased strike probability. However, shortening the deceleration duration beyond a certain threshold nearly eliminated the period of deceleration for prey. We attribute this effect on duration and strike probability to the manner in which continuous swimming increases the encounter rate between predator and prey.

**Fig. 5.**
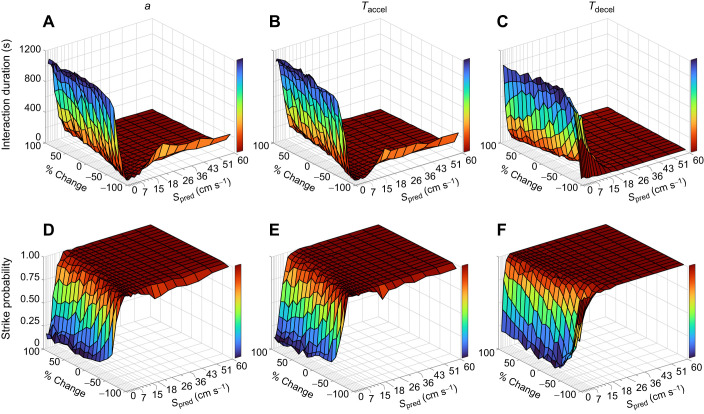
**The interactive effects of predator speed and prey swimming behavior.** Parameter values for prey swimming behavior – (A,D) acceleration magnitude (*a*), (B,E) acceleration duration (*T*_accel_), (C,F) deceleration duration (*T*_decel_) – across batches of simulations over predator speeds. The mean interaction duration (A–C) and mean predator strike probability (D–F) of individual batches are depicted on the vertical axis. Each node on the surface is the mean value of a batch of 150 simulations and the color of each polygon is determined by the value of the first vertex (the lower left corner), according to the colormap. Parameter values of predator speed were sampled along an exponential scale and thus the surface area of the polygons increases with predator speed.

These findings indicate that differences in prey swimming are most critical when the predator is moving slowly and that differences in prey speed become overwhelmed by a rapid predator. As predator speeds increased beyond the range of biologically relevant values, interactions were generally brief and deadly. In this domain, extremely intermittent prey swimming (i.e. brief and low-magnitude accelerations) did offer some improvement in survival (Fig. 5A,B,D,E), indicating a positive effect from rapid changes in motion.

### Prey heading control

**Fig. 6.**
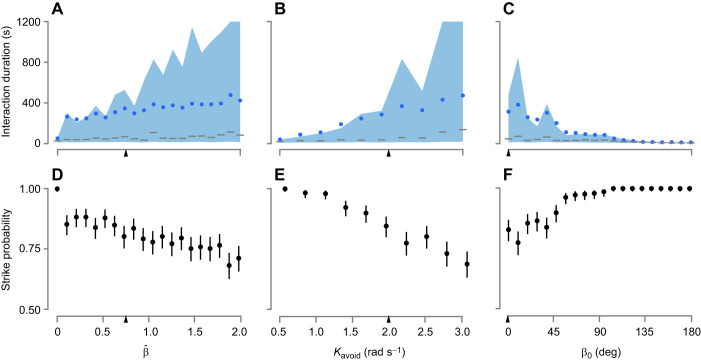
**Effects of prey avoidance strategy.** Prey avoidance strategy was examined through the values of (A,D) routine heading (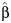), (B,E) avoidance constant (*K*_avoid_) and (C,F) avoidance heading angle (β_0_). (A–C) Shade plots depict the first and third quartiles of interaction duration, with the median (horizontal gray line) and mean (blue circles) values shown. (D–F) The strike probability (black circles) and 95% confidence intervals (vertical black lines) for the same simulations as in A–C. The simulation validated value (black triangle) of each variable is shown on the *x*-axis.

We explored the effects of spontaneous changes in heading by varying the routine heading constant 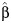. A complete lack of avoidance swimming (i.e. 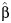
=0) showed almost instantaneous prey capture, with an ensured predator strike (Fig. 6A,D). However, increasing values for the routine heading constant permitted the direction of turns to be more influenced by the effects of predator avoidance, which gradually increased the mean interaction duration and decreased the strike probability.

**Fig. 7.**
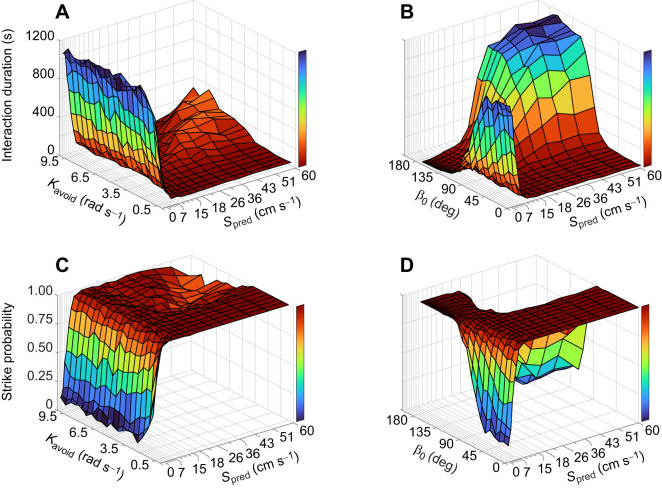
**Interactive effects of predator speed and avoidance strategy.** We examined the interactive effects of predator speed by varying parameter values for the prey's avoidance constant (*K*_avoid_) and avoidance heading angle (β_0_) for batches of simulations across predator speeds. The mean interaction duration (A,B) and strike probability (C,D) are depicted on the vertical axis. Each node on the surface represents the mean value of a batch of simulations and the color of each polygon is determined by the value of the first vertex (the lower left corner), with a colormap indicated to the right. Parameter values of predator speed were sampled along an exponential scale and thus the surface area of the colored squares increases with predator speed.

The extent to which prey avoided predators was determined by the avoidance constant *K*_avoid_, and the avoidance heading β_0_. The avoidance constant, the rate at which a prey turns away from a predator (Eqn 10), created a nearly linear increase in the interaction duration and decrease in strike probability (Fig. 6B,E). In particular, a 6-fold range in values of this parameter exhibited a nearly 90% increase in the mean interaction duration and almost a 20% decrease in the strike probability. Values of β_0_ from 0 to 45 deg resulted in the longest duration (Fig. 6C) and the lowest mean strike probability (Fig. 6F), with greater angles in that range corresponding to a rapid decline in mean interaction duration and elevated strike probability. Increasing values of the avoidance bias angle beyond 45 deg showed only small effects on the brief and deadly interactions with the predators. The effects of *K*_avoid_ largely held across the range of predator speeds (Fig. 7A,C). Therefore, the responsiveness related to turning away from the predator's heading did succeed in aiding in the avoidance of a slow predator. As predator speed increased, only extremely high values of the avoidance constant caused an increase in the interaction duration and a corresponding decrease in the average strike probability.

We found that the direction of predator avoidance that was most successful depended on the speed of the predator. This interaction created four distinct regions that included two strategically optimal regions in parameter space. Intuitively, prey were more effective when moving away from predators (0 deg≤β_0_≤90 deg) that moved slowly (i.e. speeds comparable to lionfish, *S*_pred_≤6 cm s^−1^). This directional strategy failed at higher speeds (Fig. 7B,D). However, for rapid predators (i.e. much faster than lionfish: *S*_pred_≥6 cm s^−1^), prey fared best when they directed their swimming toward the predator (90 deg≤β_0_≤180 deg).

## DISCUSSION

Our model provides a basis for evaluating the locomotion of prey fish when attempting to avoid encounters with predators. Its predictions successfully replicated the kinematics of predator–prey interactions between lionfish (*Pterois volitans*) and damselfish prey (*Chromis viridis*). In particular, we were able to validate populations of simulations against the results of previous experiments (Peterson and McHenry, 2022). Using the validated model, we identified optimal avoidance strategies for prey encountering predators operating across a wide range of speeds. The results of our sensitivity analysis contrasted the predictions of classic models of escape strategy, which assume simplified motion and predators that are faster than their prey (Isaacs, 1965; Weihs and Webb, 1984; Domenici, 2002; Kawabata et al., 2023; Soto et al., 2015).

**Fig. 8.**
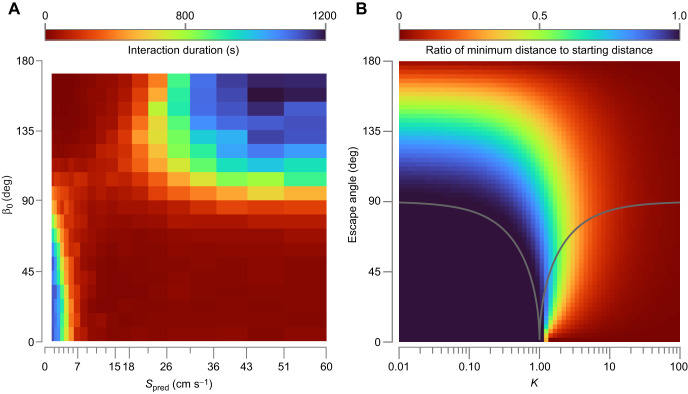
**Comparison of avoidance and evasion models of prey strategy.** (A) The performance of avoidance behavior is presented by the mean interaction duration (colormap) with respect to predator speed and the prey's avoidance heading constant, β_0_ (a 2D view of Fig. 7B). (B) Escape performance is shown by the minimum distance (colormap) between predator and prey, normalized by distance at the start of an escape (modified from Soto et al., 2015), with respect to the escape angle of prey and the relative predator speed (*K*). The optimal escape angle (in gray) is plotted for each predator speed.

The optimal strategy for prey fish is similar whether prey avoid or escape from a relatively slow predator. If the prey's routine swimming speed exceeds that of the predator, then the prey should, unsurprisingly, move away from the predator. Although the logic of this strategy would appear self-evident, the specific differences between avoidance and escape are noteworthy. In our modeling, the avoidance heading is effectively a set-point for the control of locomotion, where moving away from a predator is achieved over a range of angles (0 deg≤β_0_≤90 deg). This strategy is only effective for a narrow range of predator speeds and there is a substantial decline in interaction duration for moving in any other direction (Fig. 8A). We compared these predictions against distance-optimal escape models that assume a fixed heading for both fish in the period after a single escape response (Weihs and Webb, 1984; Soto et al., 2015). Compared with our avoidance model, it is similarly beneficial to escape away from a slow predator, though directions slightly toward the predator (90 deg<β_0_≤135 deg) incur only a slight distance penalty compared with the optimum achieved when heading away from or perpendicular to the predator (Fig. 8B). Therefore, an escape response can be effective over a more broad range of directions than what is exhibited for avoidance swimming when encountering a slow predator.

The distinction between avoidance and escape is substantially more divergent for prey challenged by a fast-moving predator. The direction of an escape is most meaningful when the predator moves at a speed similar to the prey, or up to 10 times faster, with the optimal escape direction varying with the relative speed of the two fish (Fig. 8B). For predators moving more than an order of magnitude faster than a prey, this optimum escape angle converges on a direction that is perpendicular to the predator's heading, though the distance advantage offered by this optimum is small relative to other directions. Therefore, the direction of a prey's escape matters little if they are much slower than the predator.

In contrast, we found that the optimal strategy for avoidance was to move toward the faster predator (135 deg≤β_0_≤180 deg). This strategy yielded comparable outcomes to the optimum when encountering a slow predator (Fig. 8A). A strategic change in direction toward the predator is effective because of the more limited capacity of the predator to change direction. Therefore, a predator has insufficient time to adjust its heading for a strike when an approaching prey rapidly closes the distance between the two fish. In contrast, avoidance swimming away from a fast predator is ill fated because the predator can easily overtake a prey in its attempt to move away from the threat.

Our finding of an optimal avoidance strategy for fast predators bears some similarity to other models of predator–prey interactions. Martin et al. (2022) found that fish directing an escape towards a fish predator can yield distance-optimal results when accounting for observed limits to rates of rotation and latency in the response. In this escape model, prey exhibited greater success when their turning radius was orders of magnitude less than that of the predator. A small bird can similarly succeed by executing tighter turns to evade a more massive raptor in pursuit (Howland, 1974). This turning-gambit model largely explains the ability of moths to survive encounters with bats, though these prey additionally execute escape maneuvers to enhance their survival (Corcoran and Conner, 2016).

The relatively slow turning rates of predators offer prey a strategic advantage in a number of these interactions. For escapes, the prey have the additional advantage of choosing the timing of an evasive turn, whereas the predators must respond to the prey. Large predators exhibit longer sensory processing times than their smaller prey (Domenici et al., 2011; Martin et al., 2022), and a more massive body and potentially faster speed generate proportionally greater momentum. Because the centripetal force required to execute a turn varies with the square of this momentum, a predator's locomotor system must generate disproportionately greater lateral forces to turn as quickly as the prey. It is therefore perhaps unsurprising that predators generally follow a path with a higher radius of curvature than their prey (Martin et al., 2022; Corcoran and Conner, 2016; Howland, 1974). The turning of lionfish is likely also affected by how they flare their large fan-like pectoral fins (Peterson and McHenry, 2022; Green et al., 2011; Sarhan and Bshary, 2021). Our model does not parse the effects of mechanics and neurophysiological latency, but rather encapsulates their effects through a tracking constant (*K*_track_) and an avoidance constant (*K*_avoid_), which dictates the rate at which the predator and prey respond to one another.

There are a number of strategic considerations that are beyond the scope of our model. For example, the large frontal view imposed by the pectoral fins of lionfish may induce a greater looming stimulus to prey at higher speeds (Dill, 1972; McKee and McHenry, 2020; Thompson et al., 2023), which has been shown to elevate the distance at which prey display evasive behaviors (Cooper, 2005, 2006; Cooper et al., 2009). This endows the lionfish with a body that should generate substantial drag, though the fin flaring likely allows them to limit the range of escape routes available to prey (Thompson et al., 2023). As the drag, and hence energetic cost, of locomotion increases with the square of speed, the lionfish may be operating at a speed that balances the energetic cost of locomotion with the energetic gain of capturing prey. Although any slight decrement in speed would catastrophically decrease their likelihood of capturing prey (Fig. 3A,B), moving any faster may not be worth the costs. Additionally, considerations of predator avoidance commonly address trade-offs between the safety of a refuge and missed opportunities for activities, such as foraging, that are essential for prey growth and reproduction (Ydenberg and Dill, 1986). Such energetic and ecological trade-offs remain important avenues for future exploration to further contextualize predator–prey interactions.

In summary, our agent-based avoidance model represents a significant departure from classic escape models by explicitly simulating the interactive behaviors of both predator and prey for the duration of their interaction. This contrasts a focus solely on single prey escape responses and does not require an assumed fixed heading, as in prior escape models (Isaacs, 1965; Weihs and Webb, 1984; Domenici, 2002; Soto et al., 2015; Martin et al., 2022). This distinct approach allowed us to evaluate predator–prey interactions within a dynamic framework, identifying optimal prey strategies that differ notably from predictions of traditional models.

We found distinct differences in the optimal strategy prey should adopt when encountering both slow and fast predators. Prey can successfully avoid slower predators by moving across a range of directions, from directly away from to perpendicular to the predator. However, moving in a direction toward the predator may only be moderately successful when eliciting an escape response. When prey incorrectly move toward a slow fish predator, like lionfish, they risk capture by effective suction-feeding strikes. Lionfish routinely capture prey that are within 7 cm of their mouth, a significant distance for a 3.5 cm long prey fish (Peterson and McHenry, 2022). In contrast, we found that the optimal avoidance strategy for prey encountering very fast predators was to move toward the threat, a strategy that directly contrasts the predictions of distance-optimal evasion models (Fig. 8). Our model highlights the significance of predator maneuverability constraints, such as limited turning rates due to greater momentum and morphological characteristics, which confer strategic advantages to agile prey, even during avoidance swimming. Thus, our results underscore how integrating agent-based dynamics for both predator and prey can yield novel insights into behavioral strategies and interaction outcomes beyond those predicted by traditional theory.

## Supplementary Material

10.1242/jexbio.249595_sup1 Supplementary information
