# Supplementary material for: Optimal locomotor strategy for predator avoidance in fish prey
Source: J Exp Biol. 2026 Jun 12;229(11):jeb249595. doi: 10.1242/jeb.249595 (PMC13286347; doi:10.1242/jeb.249595)
Supplement: Supplementary information [file jexbio-229-249595-s1.pdf]

Table S1. Table of symbols

Variable	Definition
d_{strike}	Strike distance
K_{avoid}	Avoidance constant
K_{track}	Tracking constant
K_{turn}	Wall turning constant
K_{wall}	Wall avoidance constant
$S_{\max}$	Maximum prey speed
T_{accel}	Acceleration duration
T_{decel}	Deceleration duration
α	Angle of the line-of-sight
β_0	Avoidance heading angle
μ	Mean
μ_{pop}	Population mean
σ	Standard deviation
σ_{pop}	Population standard deviation
τ	Decay constant
$\theta_{\text{tan},0}$	Wall bias angle

Table S2. Fixed parameter values for simulations.

Parameter	Units	Predator	Prey
τ	s	–	2
K_{wall}	rad s^{-1}	1000	1000
K_{turn}	m^{-1}	10	10
$\theta_{\text{tan},0}$	rad	0.52	0.17
β_0	rad	–	0.01
$S_{\max}$	m s^{-1}	–	0.40
K_{track}	rad s^{-1}	1.0	–
K_{evade}	rad s^{-1}	–	2.0

Journal of Experimental Biology • Supplementary information

Table S3. Probability distribution statistics for random parameter values.

		μ_{pop}		σ_{pop}			
parameter	units	μ	σ	μ	σ	min.	max.
a	m s^{-2}	0.0394	0.0244	0.021	0.0158	0.0052	0.0935
d_{strike}	m	-2.7732	0.4090	–	–	0.0302	0.1693
T_{accel}	s	0.9985	13.65	0.6646	0.3876	0.9985	0.7280
T_{decel}	s	1.0447	0.6918	0.7426	0.4572	0.0667	2.6409

Journal of Experimental Biology • Supplementary information

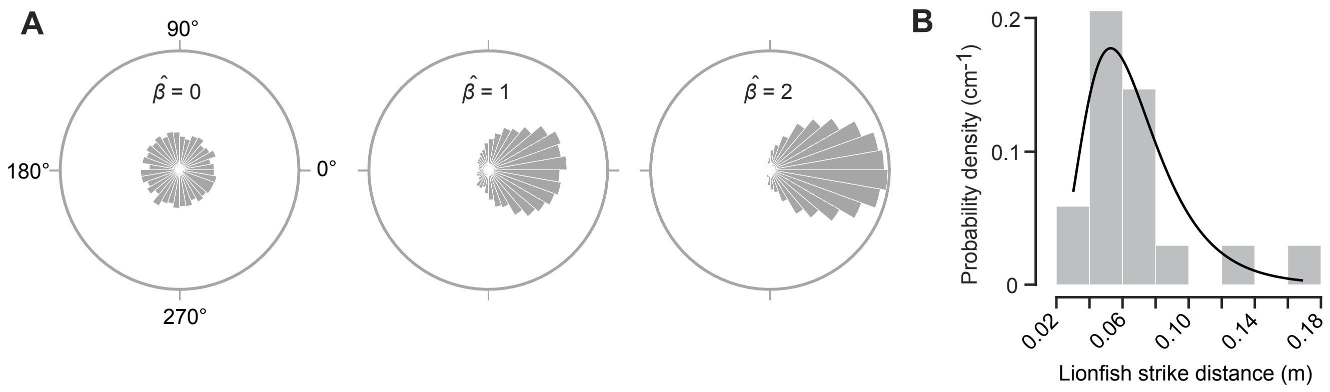

Fig. S1. Predator and prey attributes. (A) An example of the circular probability distri-bution used to determine the value of $\hat{\theta}$ which influenced changes in prey's routine heading. The shape of the circular distribution was dictated by the routine heading constant, $\hat{\beta}$, and represents the frequency that a particular angular value for $\hat{\theta}$ chosen. Values were determined by circular random number generation during at the start of acceleration and deceleration phases using the 'circ_vmrnd' function in MATLAB (Berens, 2009). (B) Histogram of measured lionfish strike dis-tances from (Peterson and McHenry, 2022), with a log-normal distribution curve-fit to the data (black line). Predator strike distance was randomly generated from this distribution at the start of each simulation.

Journal of Experimental Biology • Supplementary information

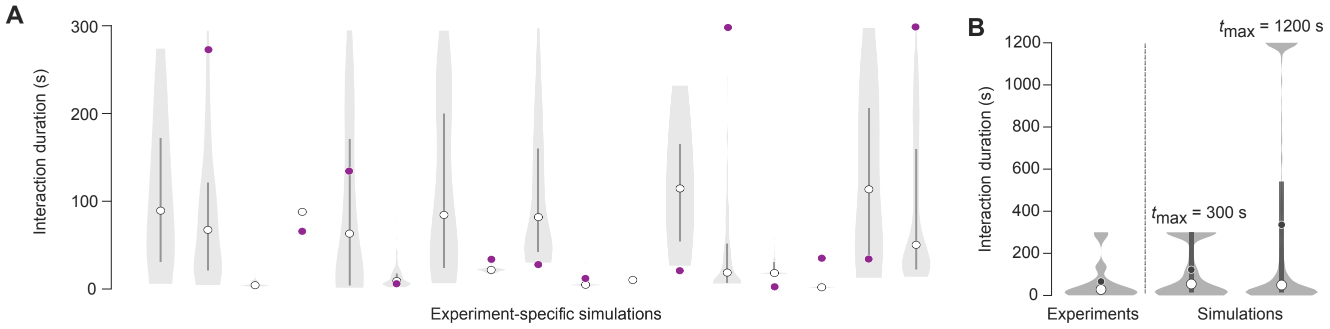

Fig. S2. Experiment-specific and population validation. (A) Violin plots show the results of experiment-specific validations for 17 experiments from Peterson and McHenry (2022), with a purple circle indicating the duration of each experiment. The absence of this marker indicates that the individual experiment did not end in the lionfish striking at the prey, however, experiment-specific validations consisted of batches of individual simulations that were still able to end in a simulated strike. (B) The duration of experiments and population validation simulations are depicted with violin plots, where the maximum simulation time was capped at 300 s and 1200 s. Population validation simulations were based on pooled kinematics, where some variables pulled from distributions of kinematics. (A–B) Violin plots visualize distributions that include kernel density estimate of the probability density function (shaded area) with inset boxplots (vertical line) with boundaries that depict the first and third quartiles with the mean value highlighted (white circle).

Journal of Experimental Biology • Supplementary information

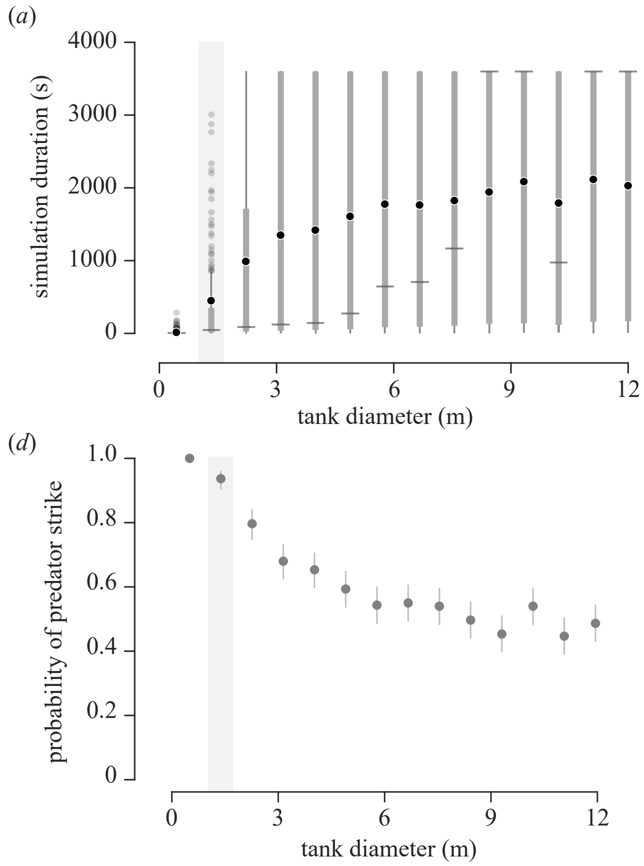

Fig. S3. Testing the effects of tank size on simulations of extended duration. The effects of varying tank diameter are shown for (a) simulation duration and (b) strike probability. Batches of 300 simualations were run for each tank size. The tank diameter was varied from 0.6 m to 12 m, with the experimental (and simulation) tank diameter of 1.2 m highlighted by a grey vertical box. Simulations were allowed to run for a maximum of 4000 s. (a) The results for simulation duration are shown with the box plots (thick vertical gray line) depicting the first and third quartiles, whiskers (thin vertical line) indicating the range of the data, outliers (gray circles), the median value (horizonal gray line), and the mean (black circles) are shown for each tank size along the x-axis. (b) The probability of a predator's strike (gray circles) for each tank size, with 95% confidence intervals (vertical grey lines).

Journal of Experimental Biology • Supplementary information

Bibliography

Berens, P. (2009). CircStat : a MATLAB toolbox for circular statistics. *J. Stat. Soft.* **31**.

Peterson, A. N. and McHenry, M. J. (2022). The persistent-predation strategy of the red lionfish (Pterois volitans). *Proc. Roy. Soc. B* **289**, 20221085.
